# Genotypic characterization of bacterial isolates causing urinary tract infections among adults at Kiambu Level 5 Hospital, Kenya: selected extended-spectrum β-lactamase genes and biofilm formation

**DOI:** 10.1099/acmi.0.000632.v4

**Published:** 2024-02-08

**Authors:** Fredrick K. Wanja, Eric O. Omwenga, Caroline W. Ngugi, John N. Maina, John N. Kiiru

**Affiliations:** ^1^​ Department of Medical Microbiology, Jomo Kenyatta University of Agriculture and Technology, PO Box 62000-00200 Nairobi, Kenya; ^2^​ Centre of Microbiology Research, Kenya Medical Research Institute, PO Box 43640-00100 Nairobi, Kenya; ^3^​ Department of Medical Microbiology & Parasitology, SHS, Kisii University, PO Box 408-40202, Kisii, Kenya

**Keywords:** antibiofilm inhibitory effects, antimicrobial resistance (AMR), biofilm formation, extended-spectrum β-lactamase (ESβL), resistance genes

## Abstract

The menace of antimicrobial resistance affecting public health is rising globally. Many pathogenic bacteria use mechanisms such as mutations and biofilm formation, significantly reducing the efficacy of antimicrobial agents. In this cross-sectional study, we aimed to determine the prevalence of selected extended-spectrum β-lactamase (ESβL) genes and analyse the biofilm formation abilities of the isolated bacteria causing urinary tract infection among adult patients seeking Medicare at Kiambu Level 5 Hospital, Kenya. The double-disc synergy test was used for phenotypic identification of ESβL-producing isolates, while microtitre plate assays with some modifications were used for the biofilm formation test. Ten isolates were bioassayed for ESβL genes out of 57 bacterial isolates obtained from urine samples. This study found the *bla*_*TEM*_ genes to be the most prevalent ESβL type [10/10 (100 %)], followed by *bla_OXA_* and *bla_SHV_* genes at 4/10 (40 %) and 3/10 (30 %), respectively. In addition, co-carriage of *bla_TEM_* and *bla_SHV_* was 50 % lower than that of *bla_TEM_+bla*_OXA_ genes at 66.7 % among *Escherichia coli* isolates studied. Biofilm formation was positive in 36/57 (63.2 %) of the isolates tested, with most being Gram-negative [25/36 (69.4 %)]. *Escherichia coli* [15/36 (41.7 %)], *Klebsiella* species [7/36 (19.4 %)] and *Staphylococcus aureus* [7/36 (19.4 %)] were the dominant biofilm formers. However, there was no significant difference in biofilm formation among all tested isolates, with all isolates recording *P*-values >0.05. In light of these findings, biofilm formation potential coupled with antimicrobial resistance genes in urinary tract infection isolates may lead to difficult-to-treat infections.

## Data Summary

For reproducibility, if needed, raw data generated from the laboratory antibiofilm drug treatmentare provided as supplementary material, Dataset S1 and can be given upon request. The Excel file has five spreadsheets, available in the online version of this article; four contain optical density (OD) readings from an ELISA reader, while the fifth has images of bacterial culture plates. Each drug treatment spreadsheet has two tables, one showing the isolate layout on the ELISA plate and the other showing OD readings. All antibiofilm treatment analyses were triplicated, and blanks were left on the plate for negative control readings.

## Introduction

Bacterial urinary tract infections (UTIs) are a primary global public health concern affecting males and females and all age groups [1]. In the past, these infections have easily been treated with existing antimicrobials, but treatment options are less effective with the ever-increasing, (re-)emergence and spread of antimicrobial resistance (AMR) [2]. The situation has worsened with the emergence of multidrug- and extensively-drug-resistant strains [1] especially in developing countries, Kenya included, with minimal strategies employed to curb this menace [2]. The change in AMR patterns has been particularly noted against β-lactam antibiotics (e.g. ampicillin, oxacillin and cephalosporins) [2]. This class of antibiotics is critical in the empirical treatment of UTIs due to their mode of action that interrupts bacteria cell-wall formation by binding to the essential penicillin-binding proteins (PBPs) and targeting both Gram-positive and Gram-negative bacteria [3]. The AMR against these crucial antibiotics has been attributed to various strategies bacteria use, such as the carriage of extended-spectrum β-lactamase (ESβL) genes that enable them to hydrolyse the antibiotics, rendering them inactive [3]. In addition to the carriage of resistance genes, the biofilm formation abilities of bacteria, particularly those causing UTIs, may promote horizontal gene transfer amongst closely positioned bacterial cells inside the biofilm and facilitate the spread of AMR genes [3]. With this in mind, there is a pressing need to build up antimicrobial surveillance and stewardship to better understand changing antibiotic resistance trends.

To date, various studies done at the global level have documented enormous and ever-increasing distribution levels of ESβL-producing bacteria, a trend that should be of great concern globally [4]. For example, a study in Iran indicated the mass prevalence of β-lactamase genes [286 (96.3 %)] amongst the isolates analysed [5]. A recent survey in the Philippines documented almost similar prevalence results [52 (66.6 %)] of ESβL genes [4]. However, regionally, particularly at Dar es Salaam, Tanzania, the reported data of the carriage of ESβL genes was confirmed in 17 (24.3 %) *Enterobacteriaceae* isolates [6]. In Kenya, a study by Kiiru *et al*. [7] reported the carriage of ESβL genes in 278 (30 %) isolates. Even so, data on the carriage of ESβLs among UTI aetiological agents in Kenya is still limited [7]. The observation may be linked to poor epidemiological surveys and the lack of routine phenotypic and genotypic characterization of multidrug-resistant micro-organisms in developing countries [7].

Biofilms, exopolysaccharide polymers that house and shield a bacterial community from antimicrobial agents and other hostile external environmental conditions, have also been reported to afford micro-organisms more tolerance and enhance quorum sensing amongst bacteria [8]. Even though biofilms do not lead to AMR per se, to succeed in UTI treatment and halt recurrence, the hypothesis that the ability to form biofilms may enhance change in bacterial phenotypic characteristics, alter gene expression and enhance AMR calls for further research [9].

Furthermore, our understanding of the interplay between biofilms and AMR among bacteria causing UTIs worldwide is limited [9] Again, the distribution of biofilm formation genes among bacteria causing UTIs is under-investigated. This scientific gap should be seen as means to aid in the development of effective antibiofilm inhibitory strategies and improve patient disease management [10–12].

Proposed long-term solutions to halt AMR with significant antibiofilm inhibitory effects to stop biofilm establishment and dissolution of existing biofilms are therefore awaited [13]. Knowledge of the carriage of resistance genes and how to halt biofilm formation may be the ultimate solution needed to allow efficient penetration of antibiotics and host immune responses into bacterial cells, resulting in prudent antibiotic therapy [14].

The current study therefore sheds light on the prevalence of ESβLs and possible biofilm formation abilities among bacterial isolates causing UTIs among adult patients seeking Medicare at Kiambu Level 5 Hospital, Kenya.

## Methods

### Study design and site

This cross-sectional study was done in Kiambu Level 5 Hospital, Kiambu County, Kenya (1 ° 10′ S 36 ° 50′ E) (Table S1).

### Sample size

The present study involved further analysis of isolates from our previous study [15] where 57 significant bacterial isolates above the ≥100 000 c.f.u. ml^−1^ (10^4^) threshold were obtained. Therefore, the 57 UTI-positive isolates were used as our sample to determine the possibility of biofilm formation in the presence of various dosages of selected commonly used β-lactam antibiotics. Isolates resistant to third-generation cephalosporins (ceftriaxone, cefotaxime and ceftazidime) and ampicillin were screened for selected ESβL gene carriage [15].

### Molecular detection of ESβLs

#### Phenotypic detection of ESβLs

The double-disc synergy test was performed to screen for carriage of ESβL genes, which involved using discs of third-generation cephalosporins and a cephalosporin-inhibitor (clavulanic acid) antimicrobial disc as previously described [16, 17]. Test discs of third-generation cephalosporins and ceftazidime/clavulanic acid were used. On inoculated Mueller-Hinton agar at 37 °C for 24 h, the discs were kept 30 mm apart, centre to centre. A transparent extension of the edge of the inhibition zone of cephalosporin towards the augmentin disc was observed as being positive for ESβL production [15]. ESβL production is inferred when the cephalosporin inhibition zone is expanded and enlarged by clavulanic acid (>5 mm) [15]. *Escherichia coli* 25992 was used as a positive control. All tests were done in triplicates that were independent of each other to validate the findings.

#### DNA extraction

Each isolate’s DNA was used as a template in PCRs and extracted using the boiling method at 95 °C for 15 min to denature the microbe’s cell wall. A loopful of pure bacterial inoculum was scrapped from the pure culture plate and immersed into 1 ml of PCR-grade water (Invitrogen; DNase/RNase free) in a 2 ml Eppendorf tube. These tubes were then placed on a heating block and heated for 15 min to lysis bacterial cell membranes by boiling at 95 °C for 15 min in the thermal block (Twin incubator; DG 210). After cooling, the tubes were placed in a centrifuge, and the contents were centrifuged at 1400 r.p.m. for 5–6 min at 26 °C to separate the DNA from other cell extracts (BioSan LMC-3000 Centrifuge; Serial no. 90804012). The supernatant containing extracted DNA was transferred to another Eppendorf tube and stored at −20 °C, until use [18].

#### Genotypic identification of ESβL genes

Molecular screening was done by PCR to determine the carriage of selected ESβL genes (*bla*_CTX-M_, *bla*_SHV_, *bla*_TEM_ and *bla*_OXA_) as described by previous studies [18–20]. The final volume in each PCR tube was 25 µl, which included 10 µl of Qiagen master mix, 1 µl butane, 2 µl DNA, 10 µl PCR water, and 2 µl forward and reverse primer [specific to each target gene; purchased from Eurofins Genomics (see Table 1 below)]. Amplification was done using a thermal cycler (GeneAmp PCR system 9700) under the following conditions: initial denaturation at 95 °C for 2 min, annealing at 50–60 °C (depending on the primer) for 1 min, extension at 65 °C for 8 min and a single final extension step at 65 °C for 8 min for 30 cycles. Amplified PCR products were separated in a 1.5 % gel, and banding patterns were visualized under a UV gel imager.

**Table 1.** PCR amplification primers used

AMR gene	Primer name	Primer sequence	Annealing temperature (°C)	Product size (bp)	Reference
*bla_TEM_*	TEM-F TEM-R	5′-ATGAGTATTCAACATTTC CG-3′ 5′-CCAATGCTTAATCAGTGA CG-3′	50	865	[18]
*bla_SHV_*	SHV-F SHV-R	5′-TTCGCCTGTGTATTATCTCCCTG-3′ 5′-TTAGCGTTGCCAGTGYTCG-3′	50	820	[19]
*bla_OXA_*	OXA-F OXA-R	5′-ATGAAAAACACAATACATATCAACTTCGC-3′ 5′-GTGTGTTTAGAATGGTGATCGCATT-3′	62	795	[20]
*bla_CTX- M_*	CTX-M-F CTX-M-R	5′-ATGTGCAGYAACAGTAARRGTKATGGC-3′ 5′-TGGGTRAARTARGTSAACAGAAYCAGCGG-3′	60	593	[19]

#### Genetic analysis of *E. coli* Isolates

Resistance to either of the tested expanded spectrum cephalosporins was used to select six *E. coli* isolates for repetitive tandem repeats analysis. This analysis used the GTG 5-PCR method using published strategies and the primers indicated in Table 1. PCR amplification of the target DNA sequence was done at an initial denaturing step for about 1 min at 95 °C, followed by annealing at 40 °C and a final extension at 72 °C. Amplified products were separated by running in 1 % agarose gel for 1 h. Visualization of banding patterns was done using a Gelmax UV imager. Banding patterns were analysed using bionumerics Gelcompar2 software version 6.6, with cluster analysis done using the dice method based on the UPGMA method. As previously described, a correlation of ≥80 % among bacterial species was regarded as solid evidence of genetic relatedness among isolates [21].

### Biofilm formation assay

The ability of the test isolates to form biofilm in the presence of different dosages of β-lactam antibiotics was determined using 96-well microtitre plate assays with some modifications as described previously [22]. The test bacteria isolates and *Pseudomonas aeruginosa* ATCC 27853 (positive control) stored at − 80 °C in vials were first thawed and inoculated on Tryptone Soya Broth (TSB; Oxoid), then incubated at 37 °C overnight. Then, a colony was identified, picked, and inoculated unto 10 ml of TSB and was incubated at 37 °C overnight while shaking at 100 r.p.m. Then, 190 µl of TSB with the antibiotics (various concentrations) were inoculated with 10 µl of bacterial cultures and incubated at 37 °C for 48 h without shaking. Flat-bottomed 96-well microtitre plates were sealed using parafilm to prevent evaporation of the medium before incubation. After incubation, the microtitre plates were rinsed gently two or three times with 200 µl double-distilled water to remove loosely attached cells/planktonic cells. The microtitre plate was then air-dried for 1 h before adding 200 µl per well of 0.4 % crystal violet solution to the adhered cells in the microtitre plates and left at room temperature for 15 min. Excess stain was removed by rinsing the microtitre plates gently with 200 µl of distilled water per well three times. The microtitre plates were then rinsed three or four times by submerging them in a tub of water, shaken out, and blotted on a stack of paper towels to rid the plate of all excess cells and dye. The plates were turned upside down and then air-dried again for 1 h. After air drying, 200 µl of absolute ethanol was added to each well to solubilize the crystal violet dye for 10–15 min. Absorbance was measured at an optical density of 630 nm (OD_630_) using a Safire Tecan-F129013 Elisa auto micro plate reader (Tecan) to determine whether an isolate is a biofilm-former or not using ethanol as the blank. The experiment was performed in triplicate using the criteria of Stepanovic *et al.* [23] where by average OD values were calculated for all the obtained 57 bacteria isolates, and the negative controls. Using the mean OD value of the negative control, the second cut-off value was established using the three standard deviations (sd) above the mean OD of the negative control (ODc), i.e. ODc=average OD of negative control + (3×sd of negative control). The final OD value of a tested isolate was expressed as the average OD value of the isolate minus the ODc value (OD=average OD of the isolate – ODC). If a negative value was obtained it was presented as zero (0), while a positive value was indicating biofilm formation. Biofilm formation was classified into strong (+++ or 3), moderate (++ or 2), weak (+ or 1) and non-biofilm producers (0) [23]. Then, antibiofilm formation inhibitory activity was performed on eight selected bacterial isolates based on their high resistance profiles against most of the test antibiotics in our previous study (*E. coli* 3323, *E. coli* 5810, *Klebsiella* sp*.* 3306, *Klebsiella* sp*.* 3063, *Staphylococcus* sp*.* 3309, *Staphylococcus* sp*.* 3328, *Proteus* sp*.* 3346, and *Proteus* sp*.* 3268 – codes as given in our laboratory) [15]. The treatment drugs used for the antibiofilm formation inhibitory activity assay were in different dosages/concentrations as follows: ampicillin (AMP) − 500, 250, 125 and 62.5 mg ml^−1^; ciprofloxacin (CIP) − 500, 250, 125 and 62.5 mg ml^−1^; sulfamethoxazole (SXT) − 960, 480, 240 and 120 mg ml^−1^; and ceftriaxone (CRO) − 1000, 500, 250 and 125 mg ml^−1^. The test drugs were purchased locally (Regal Pharmaceuticals) in different dosages, and to ensure the right dosage was used, the antibiotics were weighed on a digital weighing machine. The right dosage, e.g. 250 mg in powder, was dissolved in 1 ml of double distilled water ready for inoculation into the 96- well plates. The, 100 µl of the prepared antibiotics was dispensed into the microtitre wells with 10 µl of culture cells. The plates were then incubated for 48 h, and the staining procedure was done as previously explained. Background staining of each experiment was corrected by subtracting the crystal violet bound to un-inoculated controls from those of the samples using the formula of Omwenga *et al.* [24]. The experiments were done in triplicates that were independent of each other, and average values were calculated. The following equation was used to estimate an antibiotic’s antibiofilm activity (Abf A) [24].

Abf A (%)=(1−(ODTest sample− ODBlank)/(OD Untreated sample−OD Blank)×100.

### Data analysis

The study findings were later entered into Excel spreadsheets for analysis using GraphPad prism (version 8). Means are presented ±sem as all tests were carried out in triplicates that were independent of each other. Dunnett’s multiple comparisons ANOVA test was applied for antibiofilm formation inhibitory assays. Binary logistic regression analysis was carried out to generate the adjusted odds ratio with a 95 % confidence interval. An alpha of less than 0.05 (*P*<0.05) was considered statistically significant.

## Results

### Study population and sample size

Patient demographics are presented in the Supplementary Data (Table S1) and published in our previous manuscript [18]. Based on data from our previous study, 57/206 (prevalence of UTI, 27.6 %), isolates were used as the sample size in the current study as they had significant UTI growth [above the 100–000 c.f.u. ml^−1^ (10^4^) threshold] and were subjected to ESβL and biofilm formation analysis [15].

### Prevalence of β-lactamase phenotypes

From the 57 isolates that met the threshold for significant UTI, 14/57 (24.6 %) were resistant to ceftriaxone, cefotaxime or ceftazidime and were regarded as possible EβSL producers. However, only 10/57 (17.5 %) were confirmed as ESβL producers by the double disc method [7].

**Fig. 1.**
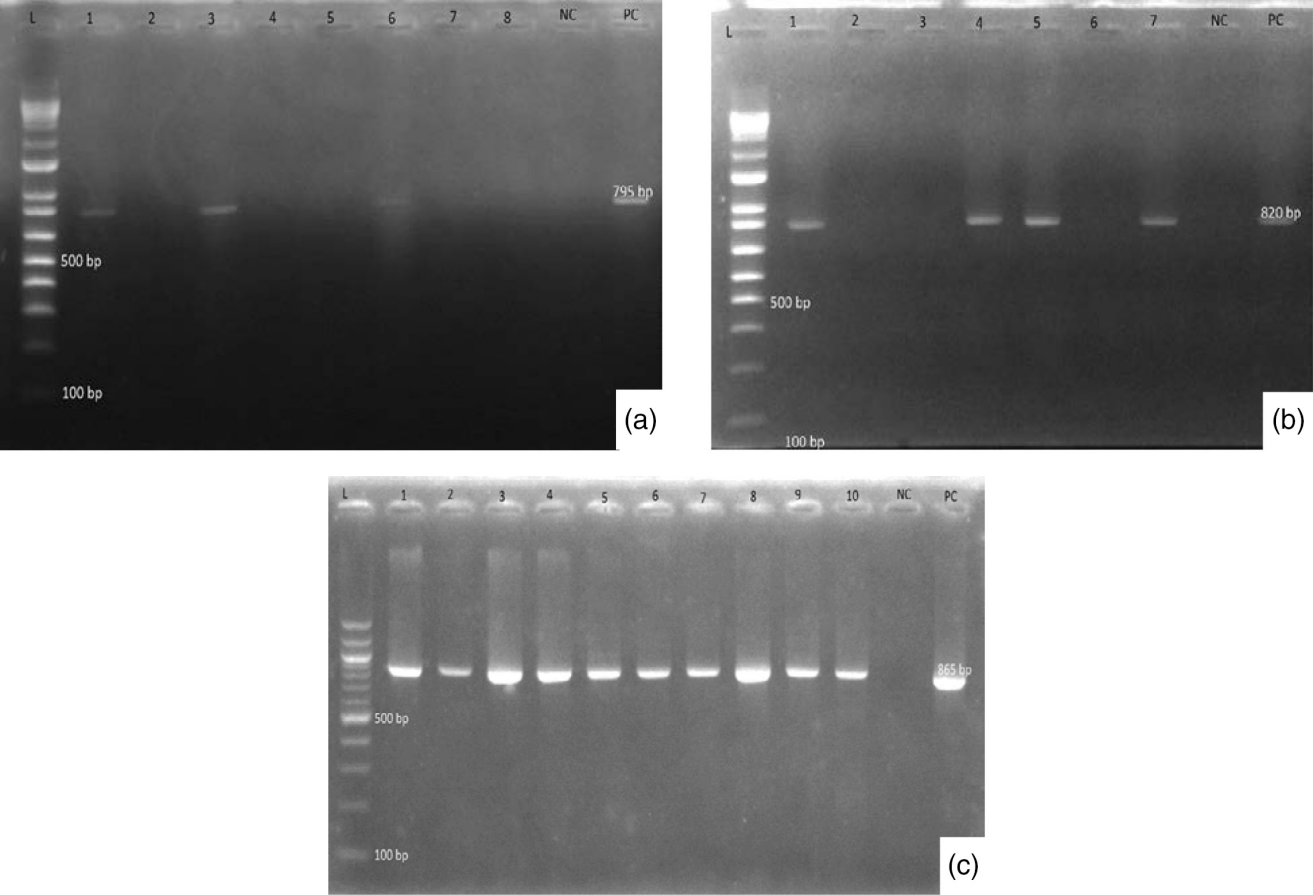
Gel electrophoresis plates for ESβL genes: *bla_OXA_* genes (a) 795 bp; *bla_SHV_* genes (b) 820 bp; and *bla_TEM_* genes (c), 865 bp. L, molecular weight ladder; NC, negative control sterile double stilled water; PC, positive control (known positive control strain). The number at the top of the plates represents random DNA numbers of the UTI isolates.

Further screening for carriage of selected *bla* genes showed that *bla_TEM_* was present in 10/10 (100 %) screened isolates and was observed at 865 bp for *E. coli, Proteus* spp., and *Klebsiella* spp*.*, as shown in Fig. 1(c). Also, *bla_OXA_* genes were present and were only detected to be present in 4/10 (40 %) of the *E. coli* isolates at 795 bp. The *bla_SHV_* genes were only present in 3/10 (30%) *E. coli* isolates at 820 bp (Fig. 1). However, the *bla_CTX-M_* gene was absent in all the isolates screened but was supposed to be observed at 593 bp (Fig. S1).

Analysis based on the co-carriage of ESβL genes revealed co-carriage of *bla_TEM_* and *bla_OXA_* at 4/6 (66.7 %) and that of *bla_TEM_* and *bla_SHV_* at 3/6 (50 %) only in the *E. coli* isolates (Table 2).

**Table 2.** ESβL gene co-carriage in bacterial isolates

Organism	*bla_CTX-M_*	*bla_TEM_*	*bla_OXA_*	*bla_SHV_*
*E. coli*	0/6 (0 %)	6/6 (100 %)	4/6 (66 %)	3/6 (50 %)
*Klebsiella* sp*.*	0/2 (0 %)	2/2 (100 %)	0/2 (0 %)	0/2 (0 %)
*Proteus* sp*.*	0/2 (0 %)	2/2 (100 %)	0/2 (0 %)	0/2 (0 %)
Total	0/10 (0 %)	10/10 (100 %)	4/10 (40 %)	3/10 (30 %)

### Genetic diversity of *E. coli* isolates causing UTIs

**Fig. 2.**
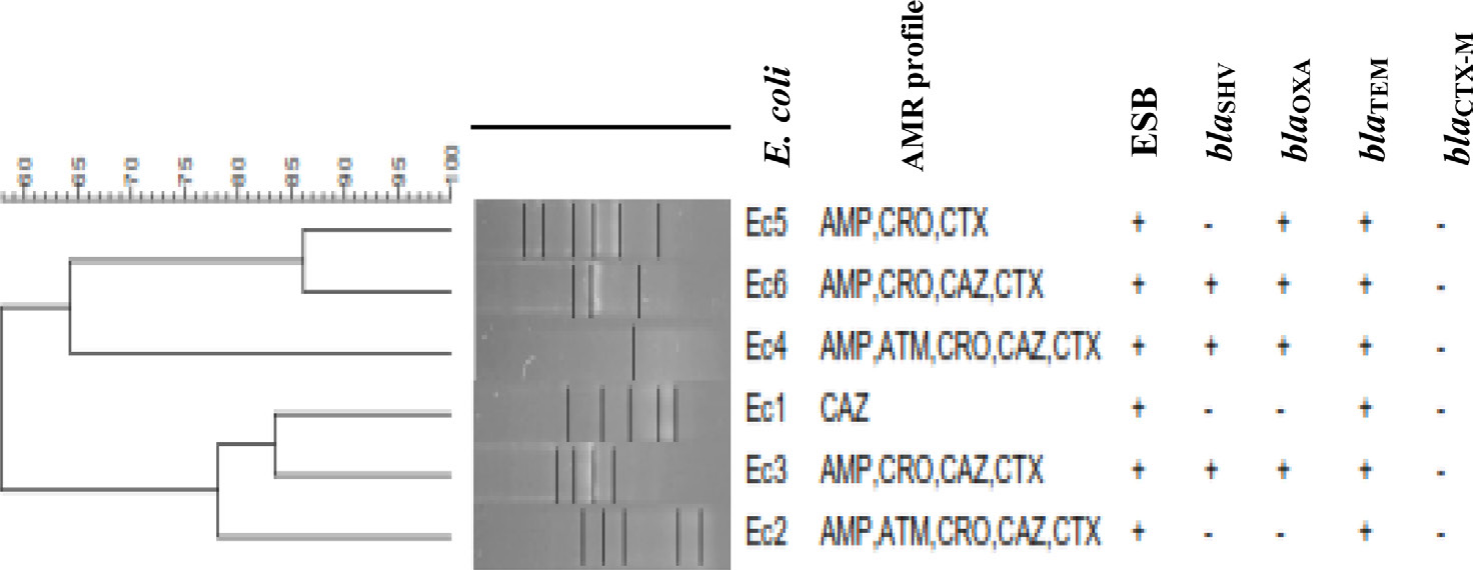
*Escherichia coli* dendrogram. The figure shows phylogentic analysis of *E. coli* isolates that were positive for carriage of EβSL genes. *bla_TEM_*, temoneria β-lactamase; *bla_CTX-M_*, cefotaxime Munich β-lactamase; *bla_SHV_*, sulhydryl variant β-lactamase; *bla*_OX_, *bla*_OXA_-type β-lactamases.

The six *E. coli* isolates, Ec1 – *E.coli* 5806, Ec2 – *E.coli* 3352, Ec3 – *E.coli* 3323, Ec4 – *E.coli* 3177, Ec5 – *E.coli* 3318 and Ec6 – *E.coli* 3350 (codes as given in the laboratory), did not cluster based on similarities in antimicrobial resistance profiles or β-lactamases gene carriage. For instance, *E. coli* 5806 and *E. coli* 3350 had identical resistance profiles (AMP, CRO, CAZ, CTX) yet clustered in different clades. This was similar to *E. coli* 3352 and *E. coli* 3323, which also had identical antimicrobial resistance (AMP, ATM, CRO, CAZ, CTX) and clustered into two clades. Therefore, the six isolates could be grouped into four clades with 65–86 % similarities, indicating no similar strain expansion in the Kiambu region (Fig. 2).

### UTI isolate biofilm formation assay

**Fig. 3.**
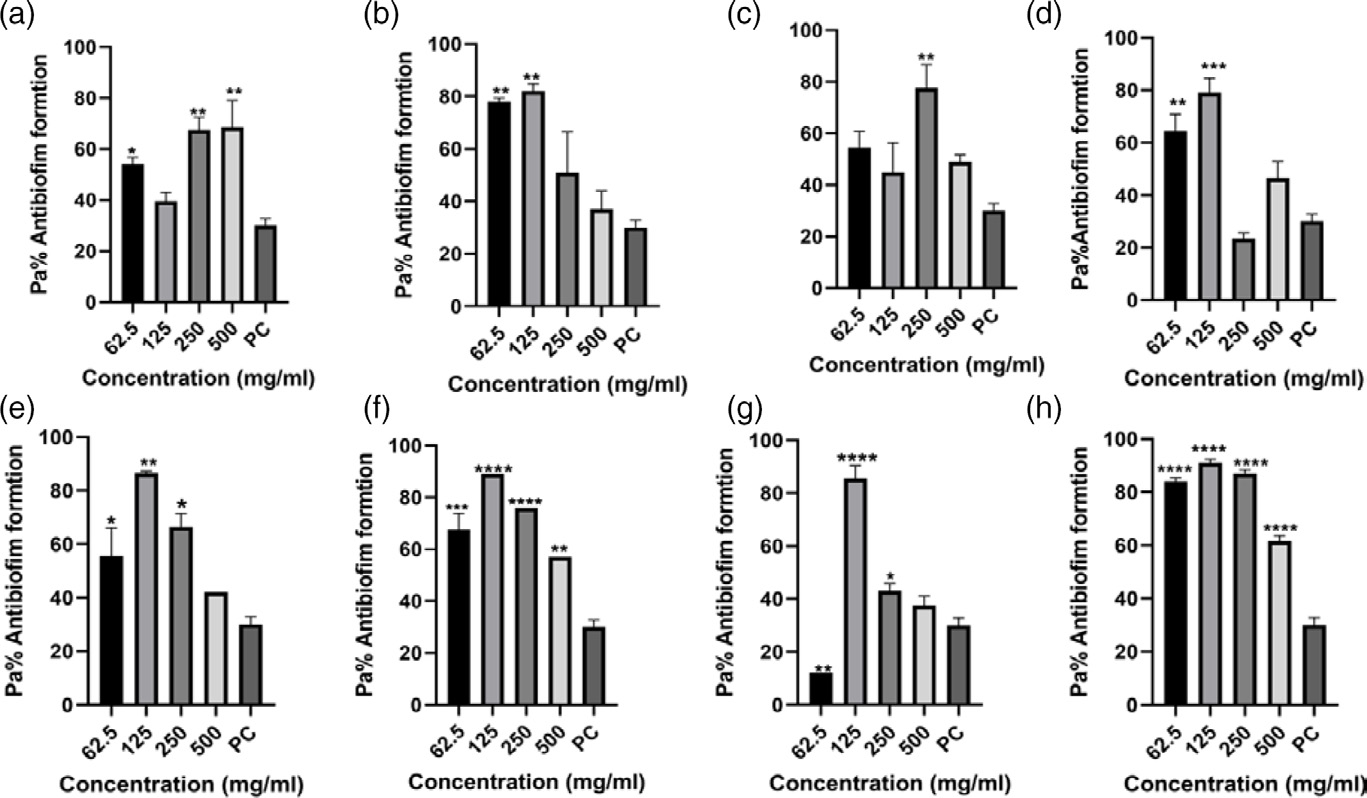
Ampicillin antibiofilm formation activity against (**a**) *E. coli* 3323, (**b**) *E. coli* 5810, (**c**) *Klebsiella* sp. 3306, (**d**) *Klebsiella* sp. 3063, (**e**) *Staphylococcus* sp. 3309, (**f**) *Staphylococcus* sp. 3328, (**g**) *Proteus* sp. 3346 and (**h**) *Proteus* sp. 3268. PC=*P. aeruginosa* – positive control (*n*=3, Dunnett’s multiple comparisons ANOVA test; *P=0.05; **P=0.01; ***P=0.001; ****P=0.0001).

**Fig. 4.**
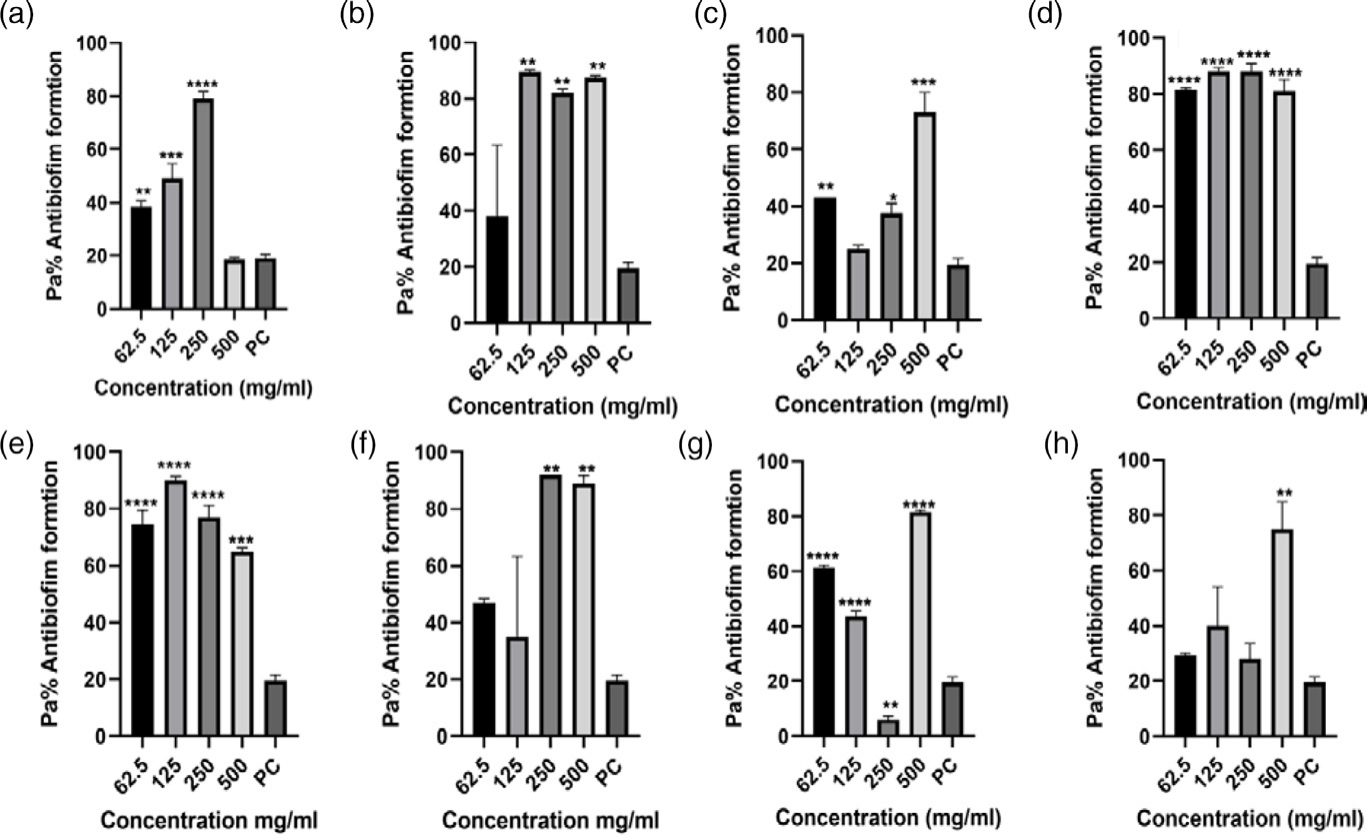
Ciprofloxacin antibiofilm formation activity against (**a**) *E. coli* 3323, (**b**) *E. coli* 5810, (**c**) *Klebsiella* sp. 3306, (**d**) *Klebsiella* sp. 3063, (**e**) *Staphylococcus* sp. 3309, (**f**) *Staphylococcus* sp. 3328, (**g**) *Proteus* sp, 3346 and (**h**) *Proteus* sp. 3268. PC=*P. aeruginosa* – positive control (*n*=3, Dunnett’s multiple comparisons ANOVA test; *P=0.05; **P=0.01; ***P=0.001; ****P=0.0001).

**Fig. 5.**
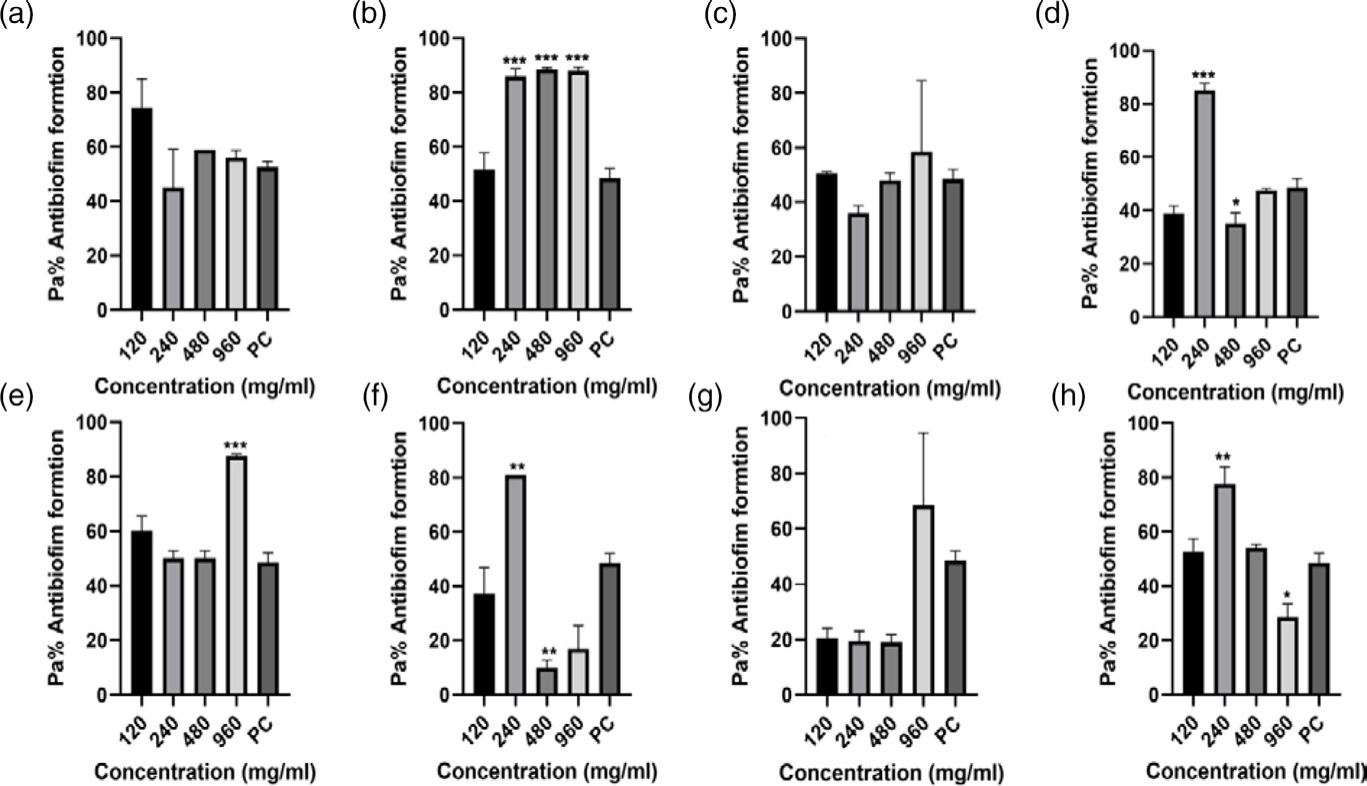
Sulfamethoxazole antibiofilm formation activity against (**a**) *E. coli* 3323, (**b**) *E. coli* 5810, (**c**) *Klebsiella* sp. 3306, (**d**) *Klebsiella* sp. 3063, (**e**) *Staphylococcus* sp. 3309, (**f**) *Staphylococcus* sp. 3328, (**g**) *Proteus* sp. 3346 and (**h**) *Proteus* sp. 3268. PC=*P. aeruginosa* – positive control (*n*=3, Dunnett’s multiple comparisons ANOVA test; *P=0.05; **P=0.01; ***P=0.001; ****P=0.0001).

**Fig. 6.**
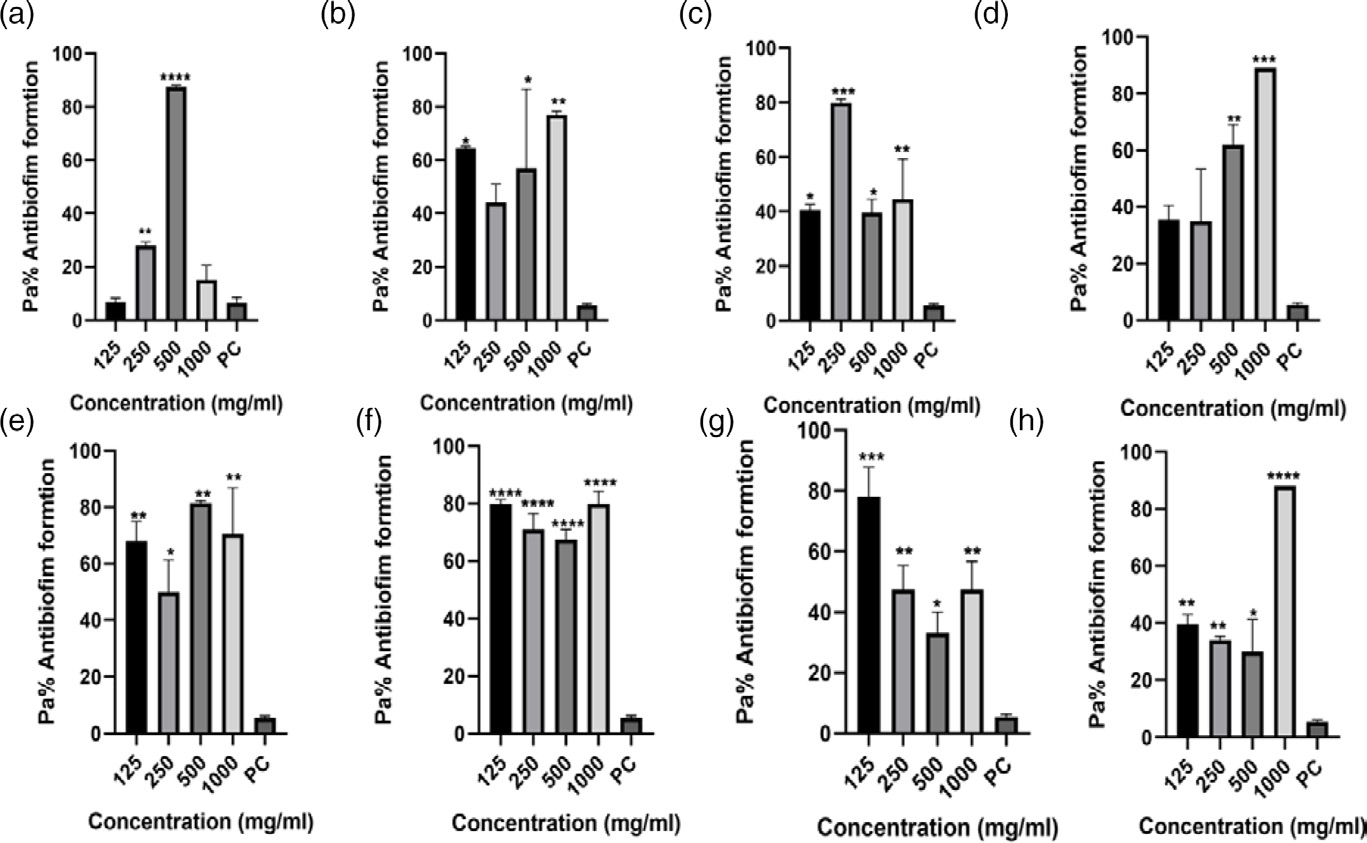
Ceftriaxone antibiofilm formation activity against (**a**) *E. coli* 3323, (**b**) *E. coli* 5810, (**c**) *Klebsiella* sp. 3306, (**d**) *Klebsiella* sp. 3063, (**e**) *Staphylococcus* sp. 3309, (**f**) *Staphylococcus* sp. 3328, (**g**) *Proteus* sp. 3346 and (**h**) *Proteus* sp. 3268. PC=*P. aeruginosa* – positive control (*n*=3, Dunnett’s multiple comparisons ANOVA test; *P=0.05; **P=0.01; ***P=0.001; ****P=0.0001).

The data from the antibiofilm inhibitory assay using four antibiotics as treatments indicated varying levels of biofilm formation inhibitory effects across the study isolates and different antibiotic dosages, compared to the positive control, as depicted in Figs 3–6. Comprehensive analysis of the antibiofilm inhibitory activity assay highlighted that ceftriaxone dosages exhibited the most notable antibiofilm inhibitory effects among all the test treatment antibiotics, as illustrated in Fig. 6. Conversely, sulfamethoxazole yielded the lowest observed antibiofilm inhibitory outcomes, as shown in Fig. 4. This analysis, iven how the classification of bacteria to the species level was impacted, demonstrated that Gram-negative isolates were more affected [69.5 %(25/36)] in comparison to Gram-positive *Staphylococcus* species that yielded significant biofilm formation ability, with a significant *P*-value of 0.0561 (Table 3).

**Table 3.** Biofilm formation analysis

Organism	Biofilm former (*N*)	Non-biofilm formers (*N*)	OR	95% CI	*P*-value
*E. coli*	15	9	1.03	0.384–2.758	0.9554
*Klebsiella* sp*.*	7	3	0.74	0.171–3.15	0.6781
*Proteus* sp*.*	3	3	1.71	0.317–9.276	0.5315
*S. aureus*	7	4	0.98	0.256–3.746	0.976
*Enterococcus* sp*.*	0	1	5.09	0.199–130.659	0.3254
*S. saprophyticus*	4	1	0.43	0.045–4.093	0.4617
Gram-negative	25	15	1.03	0.446–2.374	0.9474
Gram-positive	11	6	2.49	0.976–6.368	0.0561*

*N*, number of cases.

Nevertheless, when evaluated on an individual bacteria species basis, it was found that all treatment drugs at varying concentrations could effectively inhibit biofilm formation in *Proteus* isolates. Further analysis of biofilm formation based on patient demographics also revealed varying results (Table 4).

**Table 4.** Biofilm data analysis based on patient demographics

**Department**	**Biofilm former [36 (63.2 %)]**	**Non-biofilm former [21 (36.8 %)]**	**O. R**	***P*** **-value**	**CI**	
					**Lower**	**Upper**
Outpatient	32	17	1.86054	0.40534	0.30578	11.3632
Inpatient	4	4				
**Gender**	**Biofilm former**	**Non-biofilm former**				
Male	2/57 (3.5 %)	1/57 (1.7 %)	1.17323	0.8970091	0.05763	72.802
Female	34/57 (59.6 %)	20/57 (35.08 %)				

N, number of case.

#### Ampicillin antibiofilm formation inhibitory activity assay

Specific analysis on how ampicillin inhibited biofilm formation against the study isolates revealed significant antibiofilm formation inhibition activity against *Staphylococcus* sp*.* 3328 and *Proteus* sp*.* 3268 (***P-<0.00 and ****P<0.0001 respectively, Fig. 3). Our findings further revealed that against all the screened UTI isolates, the 62.5 and 125 mg ml^−1^ ampicillin dosages had significant antibiofilm formation activity compared to the 250 and 500 mg ml^−1^ dosages.

More analysis on how individual screened isolates were affected revealed ampicillin dosages against *Klebsiella* sp. 3306, yielding minor antibiofilm inhibition activity, with a *P*-value of (**P<0.01), especially against the 250 mg ml^−1^ dosage (Fig. 3). The raw data for this assay are given in Dataset S1, available in the online version of this article in the the first Excel spreadsheet.

#### Ciprofloxacin antibiofilm formation inhibitory activity assay

Ciprofloxacin dosages had significant antibiofilm inhibitory effects against most isolates. It was only against *Proteus* spp*.* 3268 that ciprofloxacin had a less inhibitory effect at 500 mg ml^−1^(**P=0.01, Fig. 4). Further analysis on *Klebsiella* sp*.* 3063 and *Staphylococcus* spp*.* isolates revealed significant antibiofilm inhibition activity when subjected to ciprofloxacin dosages (****P=0.0001). Moderate inhibitory activities were only observed among *E. coli* 3323, *E. coli* 5810 and *Klebsiella* spp*.* 3306 (**P=0.01 and ***P=0.001, respectively, Fig. 4).

Regarding the impact of different dosages of ciprofloxacin on the formation of biofilms by each isolate, our findings again highlighted that the 250 mg dl^−1^ dosage exhibited the most substantial inhibitory effects compared to the other dosages. The 62.5 and 125 mg ml^−1^ dosages followed closely, whereas the 500 mg ml^−1^ dosage resulted in the least inhibitory effects.

However, investigating the effects on various species, it became evident that the *Klebsiella* sp*.* 3063 isolate and *Staphylococcus* sp*.* 3309 isolate were the most influenced when exposed to different ciprofloxacin dosages. In contrast, *Proteus* species isolates demonstrated the least impact, with the *Proteus* sp*.* 3268 isolate showing no inhibitory effect when subjected to dosages of 62.5, 125 and 250 mg ml^−1^. Interestingly, the *Proteus* sp*.* 3346 isolate exhibited highly significant inhibitory effects under these dosages. ELISA optical density readings are available in Dataset S1, available in the online version of this article in the second spreadsheet of the Excel file .

#### Sulfamethoxazole antibiofilm formation inhibitory activity assay

Based on the antibiofilm inhibitory activity analysis conducted in this study, sulfamethoxazole dosages exhibited limited inhibitory effects compared to the other test antibiotics, as indicated in Fig. 5. However, a different response to sulfamethoxazole dosages was noted among *E. coli* isolates, as depicted in Fig. 5. Specifically, *E. coli* 5810 isolates could not form biofilms when exposed to sulfamethoxazole test dosages of 240, 480 and 960 mg ml^−1^, resulting in a significant *P*-value (***P=0.001).

When considering *Klebsiella* species, the analysis revealed that the *Klebsiella* sp. 3063 isolate exhibited moderate inhibitory effects in comparison to *Klebsiella* sp. 3306, which demonstrated no inhibitory effects. It is important to highlight that in the broader context, evaluating the impact of sulfamethoxazole dosages on inhibiting biofilm formation indicated that the 240 mg ml^−1^ dosage outperformed the other dosages, as depicted in Fig. 5. The raw data for this assay, i.e. ELISA optical density readings, are available in Dataset S1, available in the online version of this article in the third spreadsheet of the Excel file.

#### Ceftriaxone antibiofilm formation inhibitory activity assay

Ceftriaxone was the most effective test drug, displaying notably high or moderate antibiofilm inhibitory effects, as visually represented in Fig. 6. However, a closer examination of the individual isolates' response to ceftriaxone revealed that *Staphylococcus* species were particularly susceptible compared to the other isolates examined in this study.

Notably, the *Staphylococcus* sp*.* 3328 isolates exhibited the highest degree of susceptibility, evidenced by a significant antibiofilm inhibitory effect (****P=0.0001). Conversely, the *Klebsiella* sp*.* 3063 isolate did not display a noteworthy inhibitory effect when subjected to 125 and 250 mg ml^−1^ dosages.

It is of note that, in line with the rest of our findings, the ceftriaxone dosages also exhibited minimal inhibitory effects at the exceptionally high test dosages of 500 and 1000 mg ml^−1^, in contrast to the lower dosages of 125 and 250 mg ml^−1^, as visualized in Fig. 6.

The raw data for this assay, i.e. ELISA optical density readings, are available in Dataset S1, available in the online version of this article in the fourth spreadsheet of the Excel file.

## Discussion

The ability of bacteria to form biofilms and carriage of resistance genes may be the basis of most established and persistent UTI cases. The challenge to treat these infections may be because resistance genes such as ESβL genes may be exchanged amongst bacteria closely positioned inside the protective biofilm shields. In this study, the findings demonstrate the interplay between biofilm formation and AMR among the isolates studied. Both the current findings and those from related studies suggest that these resistance genes may underpin the occurrence of many established and persistent UTI cases [25, 26]. Carriage of *bla_TEM_* was the most prevalent, detected in 10/10 (100 %) (Fig. 1c), closely followed by *bla_OXA_* genes at 4/10 (40 %) (Fig. 1b) and *bla_SHV_* at 3/10 (30 %) Fig. 1(a). However, *bla_CTX_* was not present in any isolate screened. Based on these results, carriage of ESβL resistance genes could be the key factor that conferred resistance to the study isolates. These ESβL genes enable the bacteria of interest to break down and destroy commonly used β-lactam antibiotics. The *bla_TEM_* genotype, in particular, may be the main gene of interest as it was the commonest isolated gene. ESβLs are responsible for the increasing bacterial pathogens hydrolysing activity by enabling the breakdown of the β-lactam ring, thereby inactivating the antibiotic. Similar findings have been documented in recent related studies by [27] on how the ability to hydrolyse antibiotics by ESβL genes is causing significant resistance to various β-lactam antibiotics [28, 29].

Carriage of *bla_SHV_* and *bla_OXA_* genotypes among the study *E. coli* and *Klebsiella* spp*.* isolates was also revealed in 4 (40 %) and 3 (30 %) isolates. The findings indicate that the distribution of ESβL genes may be widespread amongst the most commonly known bacteria of medical importance. However, more surveillance research, as in the the present study, is needed to halt AMR. Such research will aid in monitoring the carriage of new emerging mutant *bla_SHV_* and *bla_OXA_* genes since most of these antimicrobial resistance genes are often encoded by mobile genetic elements (MGEs), such as self-transmissible plasmids, leading to significant change in gene expression [7]. Furthermore, it is also plausible that the prevalence of resistance genes, if not well addressed, may evolve from a narrow to an extended spectrum of hydrolysing activity, especially against monobactams and β-lactam antibiotics [30].

Significantly, strains harbouring co-carriage of ESβL genes were also identified among the isolated bacterial strains. The co-carriage of these genes may make the isolates more detrimental as they will have various resistance traits, making their management hard. These findings are in agreement with those from other related studies [31] where the test isolates expressed drug and multidrug resistance trends, particularly towards ceftazidime and cefepime, and much more against ampicillin at a range between 60 and 75 % [31]. The drug and multidrug resistance trends raise serious concerns, particularly if these strains are implicated in severe infections where limited treatment options would be available.

However, it is worth noting that the current study recorded a different finding since carriage of the *bla_CTX-M_* ESβL genotype was undetected. The *bla_CTX-M_* genotype, according to past related studies, is reported to have significant health challenges in treating community-acquired UTIs [31]. Based on this observation, screening for the carriage of all resistance genes, particularly amongst UTI causative agents, is prudent because halting the emergence and spread of new AMR genes still appears to be impossible. Health stakeholders, in particular, must accelerate education campaigns on UTI prophylactic measures to enlighten the public about the use of antibiotics in UTI treatment and assist in halting AMR.

Our phylogenetic analysis, for instance, showed significant similarity among the *E. coli* isolates at more than 70 %. The screened *E. coli* strains had identical resistance phenotypes and shared more than 75 % genetic similarity. Based on previously documented findings, there is a positive correlation between genetic diversity and the acquisition of AMR among bacterial uropathogens [32]. In this study, the observation of *E. coli* exhibiting high resistance rates against test antibiotics is of significant clinical importance, revealing that some bacterial strains may possess the same genetic similarity and still exhibit drug and multidrug resistance. Vigilant monitoring and genetic diversity assessment are thus pivotal for identifying pathogens resistant to a range of clinically meaningful antimicrobial agents [32].

Concerning the AMR trends we found, it also will be crucial to ascertain the role of biofilm formation in facilitating AMR. In this study, the isolated agents responsible for UTIs were evaluated for their capacity to form biofilms even in the presence of different dosages of antibiotics. Most isolates were capable of forming biofilms, accounting for 36/57 (63.2 %), compared to non-biofilm formers, 21/57 (35.8 %) (Table 3). However, statistical analysis indicated no significant distinction in biofilm formation, with an overall *P*-value of 0.0576. Notably, the Gram-positive isolates demonstrated an overall statistically significant tendency for biofilm formation (*P*=0.0561, Table 3). Further analysis revealed that Gram-negative isolates were the predominant biofilm formers at 25/36 (69.5 %), compared to Gram-positive isolates at 11/36 (30.5 %). Biofilm formation analysis, based on gender, showed that isolates from females exhibited a higher prevalence of biofilm formation at 34/57 (59.6 %, Table 4).

This study’s findings affirms those of past related research studies that have documented a microbial biofilm lifestyle may be aiding the pathogen’s acquisition of AMR genes that affect the static/cidal actions of antibiotics leading to a complex situation of significant health impact [10, 12, 33]. In this regard, the gains and losses of resistance genes inside bacterial biofilms need to be known to inform suitable treatment [9].

Furthermore, the biofilms allow closely placed cells to quickly transfer genes by facilitating plasmid horizontal gene exchange [34]. Other factors such as the female anatomy and indwelling intrauterine contraceptive devices may be enhancing biofilm formation by instrumentation harbouring or being reservoirs of uropathogens should be investigated [32]. These devices provide anchorage support for microbial biofilm formation and hinder easy flushing out of the attached uropathogens by urine [32]; however, more research is needed to confirm this assumption.

Therefore, the need for solutions to disrupt existing biofilms and prevent the formation of new ones is urgent. This study examined test drugs, ampicillin, ceftriaxone, ciprofloxacin and sulfamethoxazole, for their potential to inhibit biofilm formation. These drugs were chosen based on their AMR profiles, as depicted in Figs 3–6. The results of antibiofilm inhibitory activity demonstrated that ampicillin, ciprofloxacin and ceftriaxone exhibited significant inhibitory effects against the study isolates, as indicated by *P*-values above 0.05 (*). Conversely, sulfamethoxazole displayed relatively minor antibiofilm inhibitory effects. Particularly noteworthy was ceftriaxone’s pronounced antibiofilm inhibitory potential, surpassing that of the other tested drugs, as shown in Fig. 6. This heightened inhibitory effect of ceftriaxone could be attributed to its interference with mucopeptide synthesis in bacterial cell walls, hindering cell division and wall formation [9]. By contrast, the mode of action of sulfamethoxazole, involving the inhibition of dihydropteroate synthesis to impede bacterial purine and DNA synthesis, fails to prevent initial bacterial growth, thus allowing ample time for biofilm formation [25]. Though the precise mechanisms underlying how antibiotics inhibit biofilm formation have not been fully elucidated, further research on antibiofilm solutions is essential to illuminate the intricate interplay between biofilm formation and AMR.

Notably, the observed trend of lower antibiofilm inhibitory effects at higher dosages is not uncommon, and this ‘gold lock’ situation could be attributed to the possibility of aggregation of the drug at the point of cell entry that can occur at higher concentrations, reducing effective penetration [24]. This phenomenon has been reported in studies involving nanoencapsulated flavonoids against quorum sensing [24] and antibiofilm formation of *Vibrio cholerae* isolates against selected antibiotics [35]. In these cases, high dosages yielded less inhibitory impact than lower dosages [24]. This perspective suggests that antibiotics could serve as prophylactic measures against biofilm formation by uropathogens, warranting further feasibility studies for validation. However, the available antibiofilm options remain limited, presenting a significant gap in addressing this health concern. Even though several options for countering biofilm formation have been identified, such as rifampicin for Gram-positive bacteria and fluoroquinolones for Gram-negative bacteria [36], expanding the repertoire of antibiofilm solutions will facilitate more effective elimination of bacteria and bolstering efforts against the escalating trends of AMR [27, 33].

## Conclusions

According to the current study, the ultimate goal of achieving good antimicrobial stewardship and curb the rising treatment failure of UTIs could be via a holistic campaign of mapping the carriage of resistance genes that may aid biofilm formation and contribute to enhancing AMR. This will help medical care providers be equipped with the right choice of empiric treatment regimens now informed based on data of the prevailing resistance phenotypes. Besides, the data will assist in preserving the potency of β-lactam antibiotics, which are of key importance in treating most bacterial infections. The public domain will again be reassured of a health solution since there is limited hope of novel drugs.

However, from this study, the ESβL genes and the possibility of biofilm formation among the screened UTI bacteria show that there is still a gap to be addressed to achieve full success in halting AMR. Future studies focusing more on detecting other AMR genes against other classes of antibiotics and stopping biofilm formation in the first place are encouraged. Furthermore, developing novel antibiotics and compounds inhibiting biofilm formation in bacteria causing UTIs will also be a milestone achievement for modern medicine.

## Study limitations

This study had several shortcomings that can be addressed in future surveillance studies.

1. We were only able to look for a few ESβL genotypes. Future studies to determine the carriage of other β-lactamase genes are fundamental. We could also not screen for carriage of other resistance genes associated with resistance to other antimicrobial agents such as fluoroquinolones and aminoglycosides.
2. We could only determine the possibility of biofilm formation among the screened isolates. However, future studies should involve screening the carriage of genes associated with biofilms to shed light on bacterial host invasion and pathogenesis.

## Supplementary Data

Supplementary material 1

Supplementary material 2
