# Supplementary material for: Genotypic characterization of bacterial isolates causing urinary tract infections among adults at Kiambu Level 5 Hospital, Kenya: selected extended-spectrum β-lactamase genes and biofilm formation
Source: Access Microbiol. 2024 Feb 8;6(2):000632.v4. doi: 10.1099/acmi.0.000632.v4 (PMC10928394; doi:10.1099/acmi.0.000632.v4)
Supplement: Supplementary material 2 [file acmi-6-632.v4-s001.pdf]

## Table S1: Social demographic characteristics and risk-associated factors

| Variable/ statistical analysis | No. (%) obtained | With UTI | Without UTI |
|---|---|---|---|
| **Age in years** | | | |
| • ≤ 18-20 | 19/206 (9.2%) | 6/19 (31.6%) | 13/19(68.4%) |
| • 21-30 | 96/206 (46.6%) | 28/96(29.2%) | 68/96(70.8%) |
| • 31-40 | 42/206 (20.3%) | 9/42 (21.4%) | 33/42 (78.5%) |
| • 41-50 | 14/206 (6.8%) | 6/14 (42.9%) | 8/14 (57.1%) |
| • >50 | 35/206 (16.9 %) | 8/35 (22.8%) | 27/35 (72.7%) |
| • >30 years | | 23/57(40.3%) | 68/149(45.6%) |
| • ≤ 30 years | | 34/57(59.6%) | 81/149(54.4%) |
| • Odds of having a UTI >30 | | 0.4 | |
| • Odds of having a UTI ≤30 | | 0.42 | |
| • Odds ratio | | 0.953 | |
| • p-value | | 0.876 | |
| • Confidence interval | | [0.52,1.75] | |
| **Marital status:** | | | |
| • Single | 89/206 (43.2%) | 19/89 (21.3%) | 70/89 (78.7%) |
| • Married | 97/206 (47.1%) | 30/97 (30.9%) | 67/97 (69.1%) |
| • Divorced | 6/206 (2.9%) | 3/6 (50.0%) | 3/6 (50.0%) |
| • Widowed | 14/206 (6.8%) | 5/14 (35.7%) | 9/14 (64.2%) |
| • Married | | 27/57 (47.4%) | 70/149(46.9%) |
| • Single\Divorced\Widowed | | 30/57 (52.6%) | 79/149(53%) |
| • Odds of having UTI among those married | | 0.448 | |
| • Odds of having UTI among those single\divorced\widowed | | 0.38 | |
| • Odds ratio | | 1.179 | |
| • p-value | | 0.592 | |
| • Confidence interval | | [0.646,2.152] | |
| **Occupation:** | | | |
| • Employed | 78/206 (37.9%) | 28/78 (37.2%) | 50/78 (62.8%) |
| • Unemployed | 128/206 (62.1%) | 29/128 (22.6%) | 99/128(77.3%) |
| • Odds of having UTI among those employed | | 0.592 | |
| • Odds of having UTI among those unemployed | | 0.32 | |
| • Odds ratio | | 1.852 | |
| • p-value | | 0.048 | |
| • Confidence interval | | [1, 3.41] | |
| **Education:** | | | |
| • Primary | 23/206 (11.2%) | 10/23 (43.5%) | 13/23 (56.5%) |
| • Secondary | 98/206 (47.6%) | 28/98 (28.6%) | 70/98 (71.4%) |
| • Tertiary | 69/206 (33.5%) | 17/69 (24.6%) | 52/69 (75.4%) |
| • None | 16/206 (7.8%) | 2/16 (12.5%) | 14/16 (87.5%) |
| • Primary level and below | | 12/57(21%) | 27/149(18.1%) |

| | | | |
|---|---|---|---|
| • Secondary and tertiary level | | 45/57(78.9%) | 122/149(81.9%) |
| • Odds of having UTI among those with primary level and below | | 0.625 | |
| • Odds of having UTI among those with secondary or tertiary level | | 0.369 | |
| • Odds ratio | | 1.694 | |
| • p-value | | 0.155 | |
| • Confidence interval | | [0.817,3.516] | |
| **Blood Pressure:** | | | |
| • H- High | 36/206 (17.5%) | 13/36 (36.1%) | 23/36 (63.9%) |
| • L- Low | 8/206 (3.9%) | 2/8 (25.0%) | 6/8 (75.0%) |
| • Abnormal | | 15/57(26.3%) | 29/149(19.5%) |
| • Normal | | 42/57(73.7%) | 120/149(80.5%) |
| • Odds of having UTI among those with abnormal blood pressure | | 0.517 | |
| • Odds of having UTI among those with normal blood pressure | | 0.385 | |
| • Odds ratio | | 1.345 | |
| • p-value | | 0.415 | |
| • Confidence interval | | [0.66, 2.74] | |
| **Weekly sexual intercourse** | | | |
| • Once | 37/206 (17.9%) | 9/37 (24.3%) | 28/37 (75.7%) |
| • Twice | 5/206(2.4%) | 0/5 (0.0%) | 5/5 (100%) |
| • > 2 | 121/206 (58.7%) | 39/121 (32.2%) | 82/121 (67.8%) |
| • None | 43/206(20.8%) | 9/43 (20.9%) | 34/43(79.1%) |
| • Once or more | | 48/57(84.2%) | 115/149(77.2%) |
| • None | | 9/57(15.8%) | 34/149(22.8%) |
| • Odds of having UTI among those having sexual intercourse ≥ once per week | | 0.417 | |
| • Odds of having UTI among those having no sexual intercourse per week | | 0.387 | |
| • Odds ratio | | 1.078 | |
| • p-value | | 0.844 | |
| • Confidence interval | | [0.511,2.275] | |
| **No. of sexual partners** | | | |
| • One | 141/206 (68.4%) | 42/141 (29.8%) | 99/141 (70.2%) |
| • Multiple | 46/206 (22.3%) | 13/46 (28.3%) | 33/46 (71.7%) |
| • None | 19/206 (9.2%) | 2/19 (10.5%) | 17/19 (89.5%) |
| • One or more | | 55/57(96.5%) | 132/149(88.6%) |
| • None | | 2/57(3.5%) | 17/149(11.4%) |
| • Odds of having UTI among those having more than one sexual partner | | 0.427 | |
| • Odds of having UTI among those with no sexual partner | | 0.267 | |
| • Odds ratio | | 1.603 | |
| • p-value | | 0.417 | |
| • Confidence interval | | [0.509,5.045] | |
| **Frequency of changing undergarments** | | | |
| • Once | 112/206 (54.3%) | 39/112 (34.8%) | 73/112 (65.1%) |
| • Twice | 53/206 (25.7%) | 15/53 (28.3%) | 38/53 (71.7%) |
| • > 2 | 41/206 (19.9%) | 3/41 (7.3%) | 38/41 (92.7%) |

| | | | |
|---|---|---|---|
| • Once | | 39/57(68.4%) | 105/149(70.5%) |
| • More than once | | 18/57(31.6%) | 44/149(29.5%) |
| • Odds of having UTI among those changing undergarments once | | 0.371 | |
| • Odds of having UTI among those changing undergarments more than once | | 0.512 | |
| • Odds ratio | | 0.725 | |
| • p-value | | 0.327 | |
| • Confidence interval | | [0.382,1.378] | |
| **Alcohol consumption** | | | |
| • Once | 51/206 (24.7%) | 14/51 (27.5%) | 37/51 (72.5%) |
| • Twice | 26/206 (12.6%) | 10/26 (38.5%) | 16/26 (61.5%) |
| • > 2 | 44/206 (21.3%) | 6/44 (13.6%) | 38/44 (86.4%) |
| • None | 85/206 (41.2%) | 27/85 (31.7%) | 58/85 (68.2%) |
| • Yes | | 30/57(52.6%) | 91/149(61%) |
| • No | | 27/57(47.3%) | 58/149(38.9%) |
| • Odds of having UTI among those consuming alcohol | | 0.33 | |
| • Odds of having UTI among those who do not consume alcohol | | 0.545 | |
| • Odds ratio | | 0.604 | |
| • p-value | | 0.103 | |
| • Confidence interval | | [0.329,1.109] | |
| **Undergarment material** | | | |
| • Other Fabrics | 116/206 (56.3%) | 36/116 (31%) | 80/116 (68.9%) |
| • Cotton | 90/206 (43.6%) | 21/90 (23.3%) | 69/90 (76.7%) |
| • Odds of having UTI among those whose undergarment material is not cotton | | 0.45 | |
| • Odds of having UTI among those whose undergarment material is cotton | | 0.364 | |
| • Odds ratio | | 1.238 | |
| • p-value | | 0.495 | |
| • Confidence interval | | [0.672,2.279] | |
| **How frequently do you take a shower daily?** | | | |
| • Once | 139/206 (67.4%) | 38/139 (27.3%) | 101/139(72.7%) |
| • Twice | 67/206 (32.5%) | 19/67(28.4%) | 48/67(71.6%) |
| • > 2 | 0/206 (0%) | 0 (0.0%) | 0 (0.0%) |
| • Once | | 38/57(66.7%) | 101/149(67.8%) |
| • More than once | | 19/57(33.3%) | 48/149(32.2%) |
| • Odds of having UTI among those taking a shower once daily | | 0.376 | |
| • Odds of having UTI among those taking showers more than once daily | | 0.489 | |
| • Odds ratio | | 0.77 | |
| • p-value | | 0.417 | |
| • Confidence interval | | [0.409,1.447] | |
| **How frequently do you change sanitary towels during your menses (females)** | | | |
| • Once | 30/206 (14.5%) | 26/30 (86.7%) | 4/30(13.3%) |
| • Twice | 83/206 (40.2%) | 24/83 (28.9%) | 59/83 (71.1%) |
| • > 2 | 47/206 (22.8%) | 7/47(14.9%) | 40/47(85.1%) |
| • None (women in menopause and men) | 46/206 (22.3%) | 0/46 (0.0%) | 46/46 (100.0%) |

| | | |
|---|---|---|
| • Once or less | 26/57(45.6%) | 50/149(33.6%) |
| • More than once | 31/57(54.4%) | 99/149(66.4%) |
| • Odds of having UTI among those changing sanitary towels once or less daily | 0.52 | |
| • Odds of having UTI among those changing sanitary towels more than once daily | 0.354 | |
| • Odds ratio | 1.468 | |
| • p-value | 0.221 | |
| • Confidence interval | [0.794,2.714] | |

**Key word: Variable/ statistical analysis represent in N–**number of cases **%** - percentage; **%** - percentage

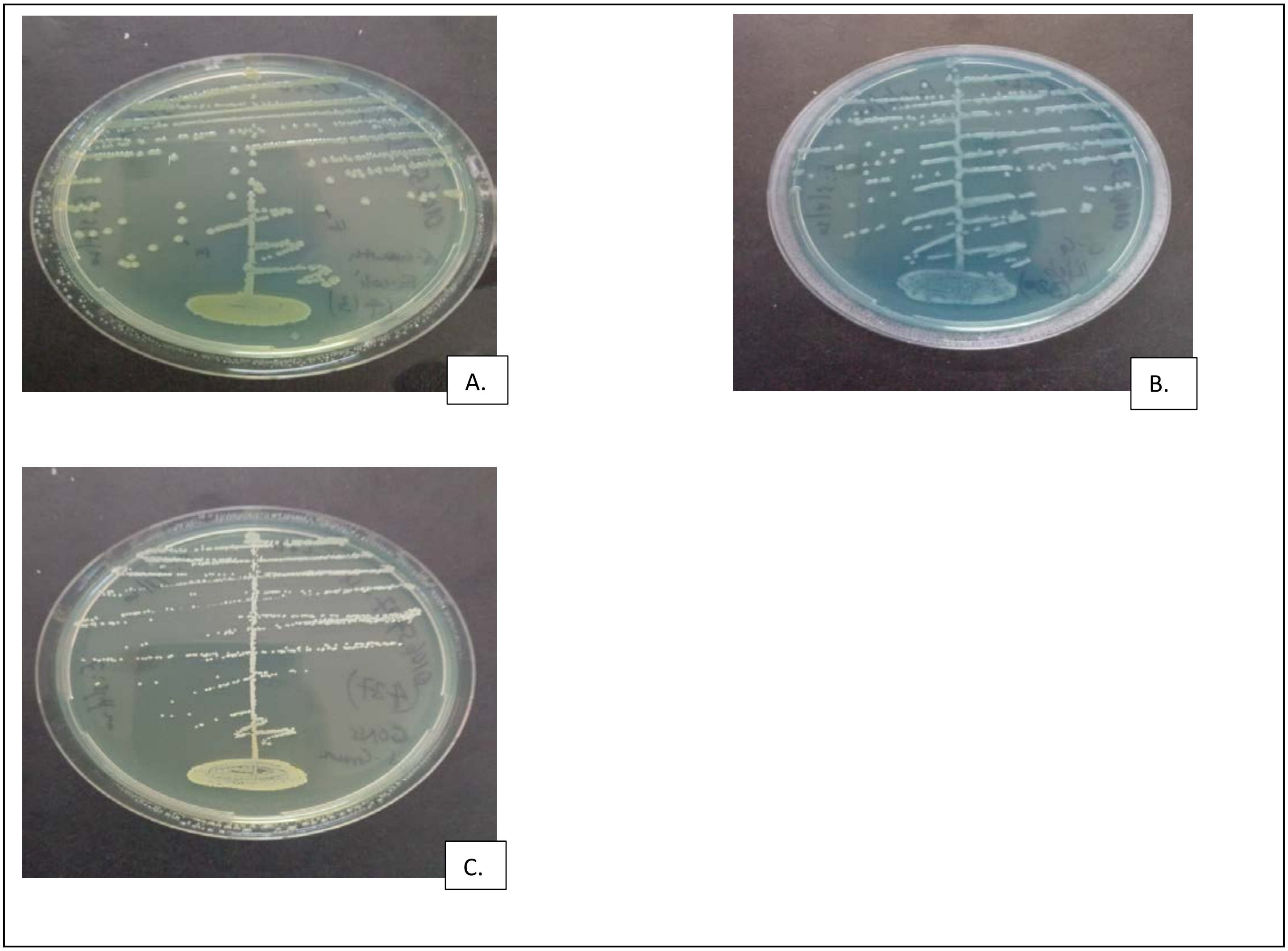


**Figure S1: Examples of the study isolate colony morphologies. Gram negative (*Escherichia coli*) A. Gram-negative (*Proteus mirabilis*) B. Gram-positive (Staphylococcus *aureus*) C.**

In text citation: The raw data for this assay are given Dataset 1, available in the online version of this article in the five Excel spreadsheet.
